# Biological and functional properties of proteolytic enzyme-modified egg protein by-products

**DOI:** 10.1002/fsn3.27

**Published:** 2013-02-14

**Authors:** Marta Pokora, Ewelina Eckert, Aleksandra Zambrowicz, Łukasz Bobak, Marek Szołtysik, Anna Dąbrowska, Józefa Chrzanowska, Antoni Polanowski, Tadeusz Trziszka

**Affiliations:** Department of Animal Products Technology and Quality Management, Wroclaw University of Environmental and Life SciencesChełmonskiego 37/41, 51-630, Wrocław, Poland

**Keywords:** Antioxidant activity, bioactive peptides, egg proteins, hydrolysis

## Abstract

Enzymatic hydrolysis led to improve functional properties and biological activity of protein by-products, which can be further used as protein ingredients for food and feed applications. The effects of proteolytic enzyme modification of egg-yolk protein preparation (YP) and white protein preparation (WP), obtained as the by-products left during the course of lecithin, lysozyme, and cystatin isolation on their biological and functional properties, were evaluated by treating a commercial Neutrase. The antihypertensive and antioxidative properties of YP and WP hydrolysates were evaluated based on their angiotensin-converting enzyme (ACE)-inhibitory activity and radical scavenging (DPPH) capacity, ferric reducing power, and chelating of iron activity. The functionality of obtained hydrolysates was also determined. Neutrase caused a degree of hydrolysis (DH) of YP and WP by-products: 27.6% and 20.9%, respectively. In each of them, mixture of peptides with different molecular masses were also observed. YP hydrolysate showed high levels of antioxidant activity. The scavenging capacity, ferric reducing power, and chelating capacity were observed at the level: 0.44 μmol/L Trolox mg^−1^, 177.35 μg Fe^2+^ mg^−1^, and 549.87 μg Fe^2+^ mg^−1^, respectively. YP hydrolysate also exhibited significant ACE-inhibitory activity, in which the level was 59.2 μg. Protein solubility was significantly improved as the DH increased. WP hydrolysate showed high water-holding capacity of 43.2. This study indicated that YP and WP hydrolysates could be used in foods as natural antioxidants and functionality enhancers.

## Introduction

Modification of food proteins in production of traditional fermented food by means of enzymatic hydrolysis has been used for many years (Liu and Chiang 2008). The consequence of proteolytic action of enzyme is a change in molecular conformation of native protein and producing functional and bioactive products that are widely used in food systems as additives for beverage and infant formula, as food texture enhancers, and as pharmaceutical ingredients (Panyam and Kilara 1996; Adebiyi et al. 2008). The properties of final products are affected by degree of hydrolysis (DH). For example, functionality changes (solubility, gelling, fat- and water-holding capacity, emulsion capacity and stability, and foaming capacity and stability) are best observed following a limited hydrolysis (Panyam and Kilara 1996). Protein hydrolysates with low DH (<10%) are used as food texture enhancers, whereas extensive protein hydrolysates are used as protein supplements or in special medical diets, such as hypoallergenic foods (Frokjear 1994; Lqari et al. 2005). In ovalbumin, removal of the 22 *N*-terminal residues (1–22) decreased its interfacial absorptivity (led to the destabilization of emulsions), whereas removal of *C*-terminal residues (346–385) increased its interfacial adsorptivity (Mine et al. 1992; Panyam and Kilara 1996). Emulsifying peptides are released during hydrolysis of αs-1 casein with chymosin (peptide: αs_1_-1–23) and β-casein with trypsin (peptide: β-1–25) (Chobert et al. 1988). Enzymatic hydrolysis with Protex (Genencor International, Rochester, NY) and Protamax (Novozymes N/A, Franklinton, NC) (DH 3% and 6%) of delipidated egg-yolk protein significantly increased the protein solubility (Wang and Wang 2009). The water-holding capacity influences the rheological properties of a system. Hydrolysis of caseins to 2.5% DH with Colorase PN or to 5.8% DH with bromelain improved water-holding capacity (Panyam and Kilara 1996).

Additional sources of edible protein hydrolysates with good functional and nutritional properties are the underutilized by-products that can be further used as protein ingredients for food and feed applications. The example of that are the fish processing by-products, delipidated egg-yolk protein produced after lecithin extraction, or by-products of the oil extraction industry, for example, sunflower and rapeseed defatted flours (Vioque et al. 2000; Wang and Wang 2009).

Protein hydrolysates from different sources, such as rapeseed, porcine myofibrillar, soy, or egg-yolk and egg-white proteins, have been found to possess antioxidant activity (Saiga et al. 2003; Gibbs et al. 2004; Yoshie-Stark et al. 2006; Xu et al. 2007). The specific activity of peptides is determined by the type and location of amino acid residues presented in their primary structure. The operational conditions employed in the processing of protein isolates, the type of protease, and the DH also affect the antioxidant activity. The main components of the antioxidative peptides are amino acids, such as tryptophan, tyrosine, cysteine, methionine, lysine, histidine, and arginine, which determine their properties (Park et al. 2001). The presence of tyrosine in the structure of peptides is especially important for the ability to scavenge free radicals. Like other phenolic compounds, this amino acid has a hydroxyl group in its aromatic ring, an electron donor to free radicals (Park et al. 2001; Davalõs et al. 2004). Antioxidant properties of egg-protein–derived peptides have been described in an increasing number of studies in recent years. Peptides, released from ovalbumin by pepsin, have been shown to have the scavenging activity (AAPH) (Davalõs et al. 2004). Also, ovalbumin was used to obtain three antioxidant peptides containing histidine, which plays a major role in the antioxidative activity, residues at the second residue in the sequence (Tsuge et al. 1991). Two peptides with aromatic residues obtained from egg-yolk proteins treated by alcalase have the potential to inhibit the oxidation of linoleic acid. A characteristic feature of these peptides, and decisive for their bioactivity, is the presence of leucine residues at the *N*-terminal position (Park et al. 2001). Hydrolysis of phosvitin with bovine trypsin leads to release the peptides capable of inhibiting the linoleic acid oxidation, scavenge DPPH free radicals, and chelate iron ions (II). These peptides are characterized by a high content of phosphorus and amino acids such as histidine, methionine, and tyrosine (Xu et al. 2007).

Phosvitin-derived peptides also increased iron uptake and showed novel antioxidant activity against oxidative stress in human intestinal epithelial cells in an in vitro assay using Caco-2 cells (Xu et al. 2007). Thus, this protein and its peptides are considered factors preventing the occurrence of oxidative stress-induced diseases such as colon cancer, Alzheimer's, or Parkinson's disease (Matoba et al. 2001).

Numerous antihypertensive peptides derived from egg proteins by enzymatic hydrolysis have been described (Miguel et al. 2004; Miguel and Aleixandre 2006). One of the best characterized a vasorelaxing peptide, ovokinin – an octapeptide (Phe-Arg-Ala-Asp-His-Pro-Phe-Leu) was isolated from the pepsin hydrolysate of ovalbumin. Ovokinin (2–7), a peptide released from ovalbumin sequence upon action of chymotrypsin, was also found to possess vasorelaxing activity. Replacement of the *C*-terminal phenylalanine by tryptophan in ovokinin (2–7) resulted in significant improvement of antihypertensive activity (Matoba et al. 2001). Miguel et al. (2004) produced, by enzymatic hydrolysis of crude egg white, two antihypertensive amino acid sequences: Arg-Ala-Asp-His-Pro- Phe-Leu and Tyr-Ala-Glu-Glu-Arg-Tyr-Pro-Ile-Leu. These peptides display inhibitory activity against dipeptidyl-carboxypeptidase (angiotensin-converting enzyme or ACE, EC 3.4.15.1), which plays an important role in controlling the development of hypertension by regulating the rennin–angiotensin system (Miguel et al. 2004; Miguel and Aleixandre 2006). Several meta-analyses revealed that drug classes have different effects on heart failure occurrence. ACE inhibitors have a better action in preventing heart failure than that of calcium channel blockers, independently of blood pressure values (Sur et al. 2011).

Three novel ACE-inhibitory peptides, IQW, IRW, and LKP, were isolated from thermolysin-pepsin ovotransferin hydrolysate. Peptide IQW was stable against digestive enzymes, suggest its effectiveness after oral administration (Majumder and Wu 2011). You and Wu (2011) reported that hydrolysates of egg-white and egg-yolk proteins treated by gastrointestinal and nongastrointestinal enzymes possessed ACE-inhibitory and antioxidant activities. Egg-protein hydrolysates produced with thermolysin and alcalase showed significantly higher ACE-inhibitory activity, whereas similar or even lower antioxidative activity, than those of hydrolysates produced with pepsin and pancreatin. Davalõs et al. (2004) reported that enzymatic hydrolysis of egg-white proteins also resulted in the production of peptides with strong ACE-inhibitory and antioxidant activities, and suggested that their combined properties would make a more useful multifunctional preparation for the prevention and control of cardiovascular diseases.

In this study, Neutrase was employed to hydrolyze two ethanol-denaturated protein by-products obtained after isolating lecithin from egg yolk and lysozyme and cystatin from egg white. The hydrolysis was performed in technical scale in the pilot plant of “Wroclaw Technological Park” to obtain functional and bioactive hydrolysates.

## Materials and Methods

### Enzyme

In this study, a commercial proteolytic enzyme – Neutrase EC.3.4.24.28 (protease from *Bacillus amyloliquefaciens*) (Sigma-Aldrich Chemie GmbH, Steinheim, Germany) – was used.

### Substrate

Two protein preparations, obtained as the by-products of bioactive egg compounds isolation, were used. The white protein (WP) preparation was received during the isolation of lysozyme and cystatin with ethanol (Sokołowska et al. 2007). The second yolk protein (YP) preparation was obtained in an ethanol extraction of lecithin from egg yolk (Siepka et al. 2010).

### Determination of protein content

The total protein (*N* × 6.25) in insoluble substrates was determined using the Kjeldahl method. Protein content in hydrolysates was determined by Lowry method (Lowry et al. 1951).

### Determination of proteolytic activity

Proteolytic activity was determined in reaction with 1% casein as a substrate (BDH, Ltd., England) at pH 7.5 (Chrzanowska and Kołaczkowska 1998). The substrate with the enzyme was incubated for 10 min at 45°C. The reaction was stopped by the addition of 5% trichloroacetic acid (TCA). The samples were then centrifuged, and the absorbance of supernatants were measured at λ = 280 nm. One unit of enzymatic activity (U) was defined as the amount of enzyme producing an increase in absorbance at 280 nm of 0.1 under reaction conditions.

Egg-protein hydrolysis was carried out according to modified method of Graszkiewicz et al. (2010).

Each substrate was mixed with distilled water at a ratio of 1:3 (w:v) and homogenized. Hydrolysis of YP and WP preparations was started by addition of Neutrase in the ratio E:S = 3:100 (v/w). The reaction was carried out at 45°C for 2 h and then terminated by heating the mixture at 95°C for 15 min. The hydrolysates were cooled, centrifuged (5500*g*, 10 min, 10°C), and the supernatants were lyophilized. Lyophilizates were stored at 4°C.

### The degree of hydrolysis

The DH (%) of the protein substrates was based on the determination of soluble peptide concentration in 5% TCA (Spellman et al. 2003).

### The content of free amino acid groups

The content of free amino acid groups (FAG) (μmol/L per g) was determined using trinitrobenzene sulfonic acid (TNBS, Sigma) according to modified Kuchroo et al. (1983) method.

### Reversed-phase high-performance liquid chromatography

Peptide profiles of hydrolysates were estimated by reversed-phase high-performance liquid chromatography (RP-HPLC). Separation was performed using a Zorbax XDB-C_18_ Agilent column (50 × 1.8 mm). The operation conditions were as follows: injection volume: 100 μL; phase A – 0.1% TFA in water; phase B – 0.1% TFA in acetonitrile, analysis time − 15 min, *T* = 30°C, flow rate 1 mL/min, gradient 0–100% B. Absorbance was measured at λ = 230 nm (Ardo and Polychroniadou 1999).

### Amino acid composition

Amino acid analysis was performed at BioCentrum Ltd. (Kraków, Poland). The protein samples were hydrolyzed in gas phase using 6 mol/L HCl at 115°C for 24 h. The liberated amino acids were converted into phenylthiocarbamyl (PTC) derivatives and analyzed by high-pressure liquid chromatography (HPLC) on a PicoTag 3.9 × 150 mm column (Waters, Milford, MA; Table 1).

**Table 1:** Amino acid composition of egg-white (WP) and egg-yolk (YP) protein preparation hydrolysates

	Composition (%)		
	
Amino acid	WP hydrolysate	YP hydrolysate	Reference for human EAA^1^
Hydrophobic amino acid			
Gly	5.83	5.56	–
Ala	8.93	8.18	–
Val^2^	7.94	6.93	1.3
Leu^2^	8.65	8.61	1.9
Ile^2^	5.63	5.29	1.3
Met^2^	3.54	2.29	1.7
Pro	4.25	4.86	–
Hydrophobic, flavor amino acid			
Phe^2^	5.21	3.91	–
Tyr	2.74	3.02	–
Hydrophilic amino acid			
Arg^2^	4.13	5.36	–
His^2^	2.04	2.19	1.6
Ser	7.97	9.21	–
Lys^2^	5.79	6.86	1.6
Asx'	10.08	10.18	–
Glx'	12.51	11.63	–
Thr^2^	4.75	5.92	0.9

Asx', Asp + Asn; Glx', Glu + Gln.

1 Suggested profile of essential amino acid requirements for adult humans, FAO/WHO (1990).

2 Essential amino acids.

### Determination of the molecular weight distribution of the hydrolysates

The molecular weights of the peptides were determined by gel filtration chromatography in a HPLC system equipped with a Zorbax GF-250 Agilent column (4.6 × 250 mm) at 30°C. An aliquot of 0.1 mL was injected and eluted using 0.02 mol/L phosphate buffer (pH 7.2) containing 0.2% NaCl at 0.5 mL/min. The absorbance was monitored at 230 nm. Bovine serum albumin (66 kDa), chicken egg ovalbumin (45 kDa), trypsinogen (24 kDa), β-lactoglobulin (18.4 kDa), lysozyme from chicken egg white (14.3 kDa), aprotonin (6.5 kDa), and bovine insulin, chain B (3.5 kDa) were used as standards.

### ACE-inhibitory activity

ACE-inhibitory activity (IC_50_) was assayed by the spectrophotometric method with the use hippury-l-histidyl-l-leucine (HHL) as substrate (Miguel et al. 2004). HHL (5 mmol/L in 100 mmol/L potassium phosphate containing 300 mmol/L sodium chloride, pH 8.3), enzyme, and peptide solutions were incubated at 37°C for 30 min. The reaction was stopped by the addition of 1 mol/L HCl. Conversion of HHL to hippurate and l-histidyl-l-leucine was quantified spectrophotometrically at 228 nm. The IC_50_ value was defined as the concentration of inhibitor required to inhibit 50% of the ACE activity (Table 2).

**Table 2:** ACE-inhibitory activity of egg-white (WP) and egg-yolk (YP) protein preparation hydrolysates

Sample	IC 50 (μg)
WP substrate	0
WP 2 h hydrolysis	245.7^a^
YP substrate	0
YP 2 h hydrolysis	59.2^b^

^a,b^The same letters indicate no statistical significant difference at *P* = 0.05.

### Determination of antioxidant activity as the ability to scavenge DPPH free radicals

Antioxidant activity was determined by a modified method of Yen and Chen as the ability to scavenge of DPPH (2,2-di(4-*tert*-octylphenyl)-1-picrylhydrazyl) free radicals in an aqueous solution of peptides (Yen and Chen 1995). Absorbance measurements were made after 30 min incubation at λ = 517 nm. The antioxidant activity of the 1 mg/mL protein solution was determined on the basis of the standard curve prepared for Trolox – synthetic antioxidant.

### FRAP method

Antioxidant activity was determined as the ability of the hydrolysate to reduce the oxidation of iron Fe(III) to Fe(II) ions in reaction with TPTZ (2,3,5-triphenyltetrazolium chloride). Absorbance measurement was made at λ = 593 nm. The concentration of Fe^2+^ ions in 1 mg/mL protein solution was determined on the basis of the standard curve for a FeSO_4_ solution (Benzie and Strain 1996).

### Determination of iron Fe(II) ion chelation

Chelation of iron ions was determined by colorimetric measurement of the quantity of Fe(II) not bounded with the hydrolysate in the reaction mixture with the participation of ferrozine (3-(2-pyridyl)-5,6-diphenyl-1,2,4-triazine-*p*,*p*′-disulfonic acid monosodium salt hydrate) (Xu et al. 2007). Absorbance measurement was made at λ = 562 nm. The ability to chelate iron ions was determined on the basis of the standard curve for a FeCl_2_ solution.

### Water solubility

Water solubility was determined by a modified method of Wang and Wang (2009). The sample was dispersed to 1% (w/v) in the pH range of 3–9 using 1 N sodium hydroxide or 1 N hydrochloric acid and heated up to 60°C for 1 h. After 12 h of incubation in 4°C, samples were centrifuged at 10,000*g* for 10 min. Protein content in the supernatant was determined using the Biuret protein assay. The calculations are shown in the following:


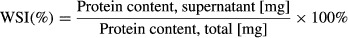


### Water-holding capacity

Water-holding capacity was determined using the centrifugation method described by Diniz and Martin (1997). Samples of 0.25 g were dissolved with 10 mL of distilled water and shaken for 30 sec. The dispersions were stored at 4°C overnight and centrifuged at 2800*g* for 30 min. The supernatant was filtrated through Whatman No. 1 filter paper and the volume of filtrate was measured as shown in Table 3. Water-holding capacity was calculated according to equation:

**Table 3:** Water-holding capacity of egg-white (WP) and egg-yolk (YP) protein preparation hydrolysates

Water-holding capacity (%)	
WP substrate	20.7
WP 2 h hydrolysis	43.2
YP substrate	6.7
YP 2 h hydrolysis	12.8


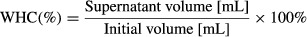


### Emulsifying activity

Emulsifying activity was determined by the turbidimetric method (Jeon et al. 1999). The solutions of protein hydrolysates (2% w/v) were acidified with acetic acid to pH 6.5. Three milliliter of solution was homogenized with 1-mL rapeseed oil (12,000 rpm, 1 min). The mixture was maintained at 20°C. A 150 μL of emulsion was mixed with 7.5 mL of 0.1% SDS, followed by absorbance measurements at λ = 500 nm at 1-min intervals.

### Data analysis

All experiments were carried out in triplicates. The data obtained were subjected to multifactor analysis of variance (ANOVA), followed by the Duncan's multiple range test to determine the significant difference between sample at *P* < 0.05 level using the Statistica v. 9.0.

## Results and Discussion

### Enzymatic hydrolysis

Hydrolysis of egg by-products was performed with a commercial enzyme Neutrase, which was effectively used to hydrolyze food proteins, such as soy proteins, gluten, or collagen (Xiangzhen et al. 2007; Siriporn et al. 2008; May-June et al. 2010). The significant increase of hydrolysis (in vitro) to release bioactive and/or functional fragments from food proteins can be observed (Spellman et al. 2003). Numerous egg-derived peptides exhibiting various activities such as anticancer, antihypertension activity, immunomodulation, mineral binding, antimicrobial, or antioxidant have been reported (Davalõs et al. 2004; Miguel et al. 2004; Mine and Kovacs-Nolan 2006).

Taking this into account, we proposed enzymatic hydrolysis as a method for utilizing ethanol-denaturated protein by-products left during the course of isolating lecithin from egg yolk, and lysozyme and cystatin from egg white. First, hydrolysis was performed to obtain antioxidant and antihypertensive peptides, and second was to enhance the functional properties of proteins.

The progress and kinetics of the hydrolysis were analyzed by monitoring the DH and free amino group content. RP-HPLC of final hydrolysates was also performed.

**Figure 1:**
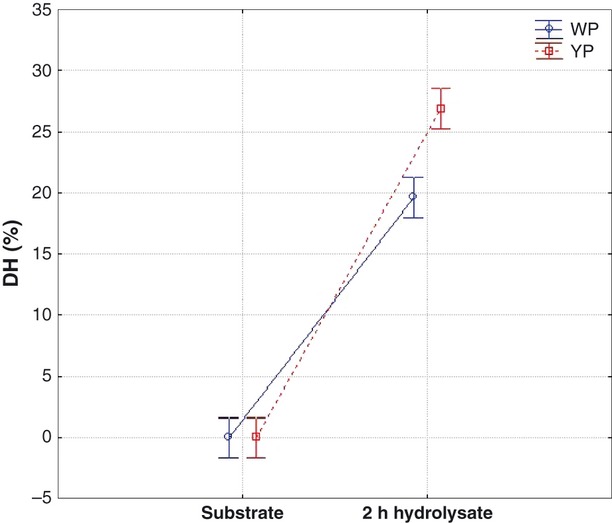
The degree of hydrolysis (DH) of egg-yolk (YP) and egg-white (WP) protein preparations treated by Neutrase.

**Figure 2:**
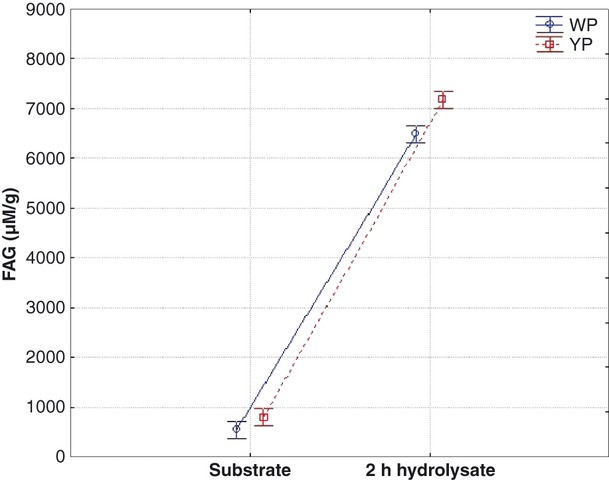
Changes in free amino groups contents (FAG) in egg-yolk (YP) and egg-white (WP) protein preparation treated by Neutrase.

The DH is an important parameter in enzymatic modification of proteins and might be a factor controlling the composition and properties of products (Ge and Zhang 1993). As shown in Figure 1, the DH of YP hydrolysate (27.6%) was higher than that of WP hydrolysate (20.9%) after 2 h hydrolysis. This is in agreement with results obtained by Miguel and others, who reported that some egg-white proteins, for example, ovotransferin, were much more resistant to proteolysis (Miguel and Aleixandre 2006). More extensive degradation of WP with various proteolytic enzymes than with Neutrase was observed by other authors (Graszkiewicz et al. 2007, 2010). The DHs of the 1-h hydrolysates of WP were 77.6%, 55.8%, and 43.3% for chymotrypsin, trypsin, and elastase, respectively (Graszkiewicz et al. 2007). On the contrary, Wang and Wang (2009) conducted controlled hydrolysis of YP for improvement functionalities by proteases from *Bacillus amyloliquefanciens* and *Bacillus licheniformis*. The DHs of obtained hydrolysates of YP were 3% and 6% (Wang and Wang 2009). However, the DH depends not only on the protein substrate and the specificity of the enzyme but also on the conditions used during hydrolysis. The progress of hydrolysis was also confirmed by determination of the free amino groups content (Fig. 2). The greatest increase in their concentration was also observed for hydrolysate of YP (7067 μmol/L Gly g^−1^). However, the concentration of the free amino groups in WP hydrolysate reached 6524 μmol/L Gly g^−1^ protein. The significant difference in protein composition and their amino acid sequences results in distinct products of protein degradation by Neutrase.

**Figure 3:**
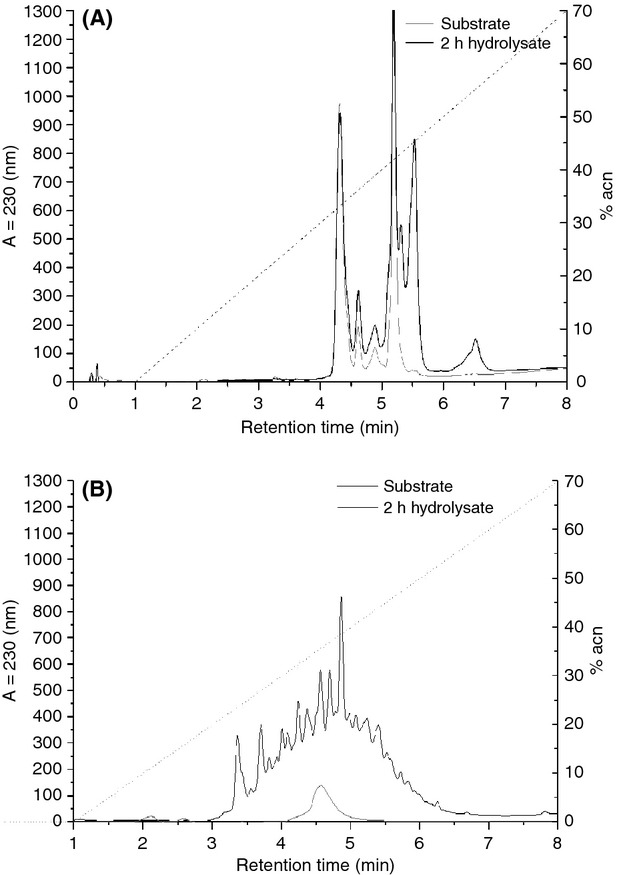
Peptide profiles of (A) egg-white (WP) and (B) egg-yolk (YP) protein preparation hydrolysates (reversed-phase high-performance liquid chromatography [RP-HPLC]).

The RP-HPLC profiles of the 2-h hydrolysates showed differences in the hydrophobicity of the generated peptides (Fig. 3A and B). All the products of the enzymatic treatment of WP and YP were eluted from 35% to 50% of acetinitrile. However, the distribution of peaks was significantly different. It confirmed the different cleavage patterns in the WP and YP as substrates by Neutrase.

**Figure 4:**
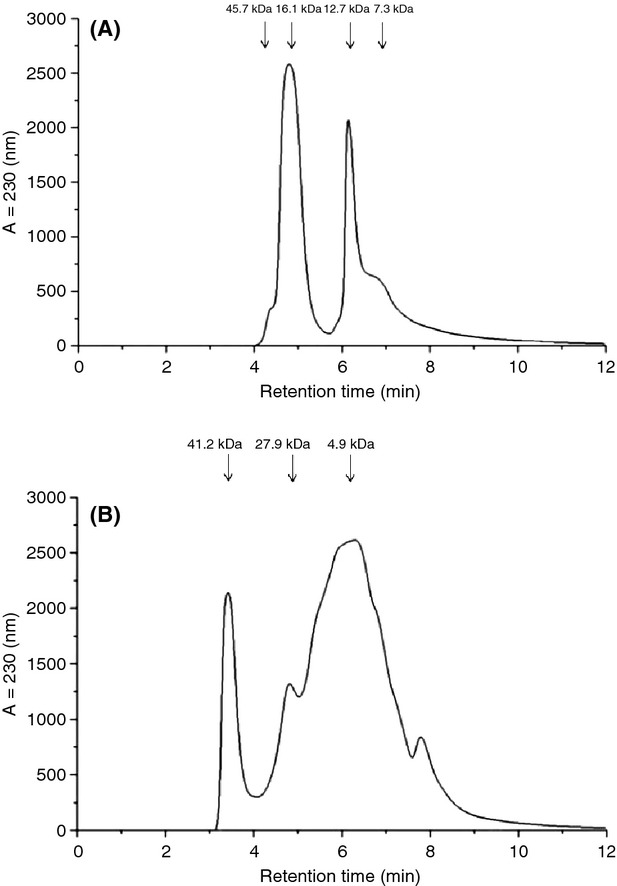
Peptide molecular weight repartition for (A) egg-white (WP) and (B) egg-yolk (YP) protein preparation hydrolysates.

The molecular weight distributions of peptides were characterized using size-exclusion chromatography. The products of degradation obtained in each hydrolysate differed in molecular mass (Fig. 4). After degradation of WP and YP, the obtained peptides exhibited molecular weights from 7.35 to 16.04 kDa and from 4.94 to 41.23 kDa, respectively.

### Angiotensin-converting enzyme (ACE)-inhibitory activity

Hypertension is one of the major risk factors for cardiovascular disease (Miguel et al. 2004). Consequently, there is the intensive development on finding effective antihypertensive agents.

The egg-protein hydrolysates treated by Neutrase had inhibitory effect on ACE activity in vitro in this study. Hydrolysate of YP showed 4.3-fold higher ACE-inhibitory activity than those of hydrolysate of WP (Table 2). The IC_50_ value of the YP and WP hydrolysates were 59.2 and 257.4 μg, respectively. Our results confirmed the observation of other authors that enzymatic hydrolysis of egg proteins resulted ACE-inhibitory peptides (Table 2) (Miguel et al. 2004, 2007). Fujita et al. (2000) demonstrated that hydrolysates obtained from ovalbumin using pepsin and thermolysin exhibited ACE-inhibitory activity. The IC_50_ values of these hydrolysates were 45 and 83 mg/mL, respectively.

The IC_50_ values of the WP and YP hydrolysates were also similar to the activity of other hydrolysates, which are reported in other studies. IC_50_ values of egg-white hydrolysates were reported to range from 9.2 to 268.6 μg/mL (Miguel et al. 2004; Majumder and Wu 2011; You and Wu 2011). Likewise, IC_50_ values of egg-yolk hydrolysates were reported to range from 52.8 μg/mL to 1.2 mg/mL (You and Wu 2011).

Extensive hydrolysis lead to obtain hydrolysates which exhibited higher ACE-inhibitory activity than hydrolysates obtained during limited hydrolysis of protein (Miguel et al. 2004). According to Hartman and Miesel (2007), ACE-inhibitory peptides are generally short-chain peptides, often carrying polar amino acid residues such as proline. However, our results showed that proline residues content did not differ significantly between the YP (4.86%) and WP (4.25) hydrolysates (Table 1).

### Antioxidant activity

Antioxidants protect human organism against the destructive activity of free radicals (oxidative stress). Antioxidants also inhibit lipid oxidation in food, which result in subsequent development of undesirable off-flavors, dark colors, and toxic reaction products (Sakanaka and Tachibana 2006). Peptides are potentially important natural antioxidants for commercial use (Simopoulos 2001; Sakanaka and Tachibana 2006). Natural antioxidant have attracted increasing interest because they are generally recognized as safe (Fang et al. 2002; Sakanaka and Tachibana 2006). Many antioxidative peptides have been isolated from natural sources, including food like those of casein, wheat gluten, soy, or egg (Chen et al. 1995; Chrzanowska and Kołaczkowska 1998; Suetsuna et al. 2000; Davalõs et al. 2004; Xu et al. 2007).

**Figure 5:**
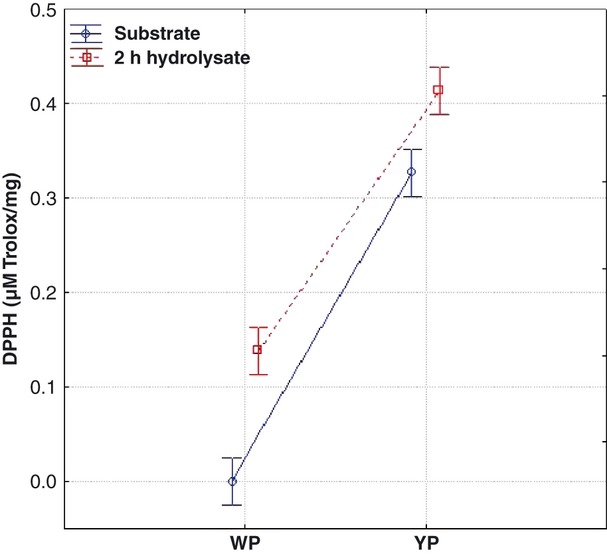
DPPH (2,2-di(4-*tert*-octylphenyl)-1-picrylhydrazyl) scavenging activity of egg-yolk (YP) and egg-white (WP) protein preparation hydrolysates.

**Figure 6:**
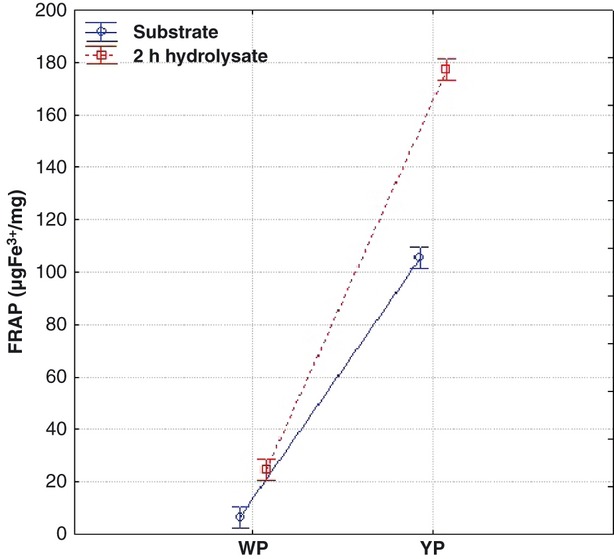
The ferric reducing ability (FRAP) of egg-yolk (YP) and egg-white (WP) protein preparation hydrolysates.

**Figure 7:**
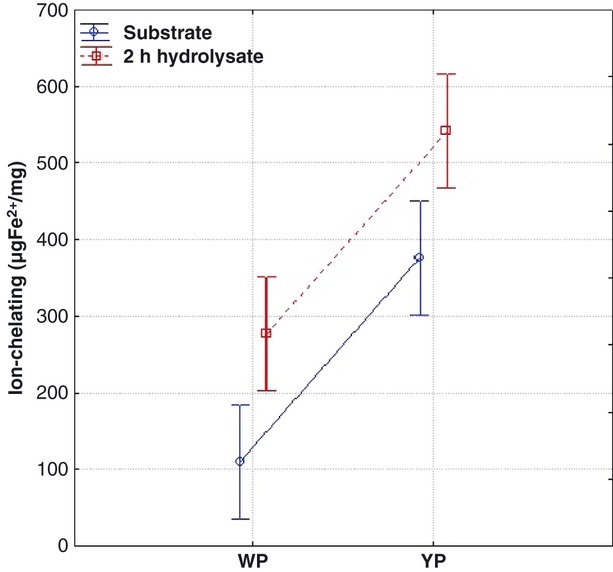
Ferrous ion-chelating activity of egg-yolk (YP) and egg-white (WP) protein preparation hydrolysates.

The antioxidative properties of the WP and YP hydrolysates were evaluated based on their radical scavenging activity with stable DPPH (Fig. 5) ferric reducing power (Fig. 6) and Fe^2+^ chelating effect (Fig. 7). In this study, the egg-protein hydrolysates demonstrated higher levels of antioxidant activity than egg protein by-products. As a result of hydrolysis of proteins, a higher number of active amino acids are exhibited, which allows their interaction with oxidizing agents (Kong and Xiong 2006). Hirose and Miyashita (1999) found that hydrolysates stand as an electron donor to free radicals and may also form a membrane protecting lipid droplets against oxidation processes. Wu et al. (2003) in their study also showed an increase in antioxidant activity as a result of progress of hydrolysis. Longtime hydrolysis of mackerel proteins leads to obtain peptides exhibited higher antioxidative activity than products of partial hydrolysis (Wu et al. 2003).

YP hydrolysate showed much higher antioxidant activity than WP hydrolysate. The levels of DPPH scavenging activity and ferric reducing power of YP hydrolysate reached 0.44 μmol/L Trolox/mg and 177.35 μg Fe^2+^ mg^−1^, respectively. Whereas the levels of DPPH scavenging activity and ferric reducing power of WP hydrolysate were 3.3-fold and 7.2-fold lower than the levels of those activities of YP hydrolysate.

YP hydrolysate exhibited a much higher DPPH radical scavenging activity than WP, probably due to their differences in amino acid composition.

It has been reported that His, Pro, and Tyr are the most important residues in radical scavenging activity of antioxidant peptides (Xia et al. 2012). YP hydrolysates contained slightly higher contents of amino acid residues than WP hydrolysates (Table 1).

The free radical scavenging activity in egg-white proteins hydrolysates was reported by other authors (Davalõs et al. 2004; Graszkiewicz et al. 2007, 2010). Graszkiewicz et al. (2010) demonstrated similar levels of free radical scavenging activity of hydrolysates of WP treated by trypsin. They isolated few peptide fractions, which had antioxidant activity values between 0.186 μmol/L Trolox mg^−1^ and 1.37 μmol/L Trolox mg^−1^. In their further study, they showed that the degradation of WP with chymotrypsin and elastase generates peptides with significantly lower free radical scavenging activity than that of WP hydrolysate treated by trypsin (Graszkiewicz et al. 2010).

Peptide fractions isolated from tryptic hydrolysate of phosvitin also exhibited strong antioxidant activity. The residual radical percentage reached 82.5% at a low concentration of fractions (Xu et al. 2007).

According to Flaczyk, protein hydrolysates from plant sources possess much higher ferric reducing activity than hydrolysates obtained from animal proteins (Flaczyk 2005). On the contrary, in this study, YP hydrolysates exhibited strong ferric reducing activity.

YP hydrolysate possessed much higher reducing power than WP hydrolysate. The phenolic and indolic groups of tyrosine and tryptophan have been reported to play important roles as hydrogen donors in a redox system (Xia et al. 2012). YP hydrolysate contained significantly higher contents of these amino acid residues than WP hydrolysate (Table 1). The contribution of tyrosine and tryptophan was 3.02 and 5.92 in YP hydrolysate. However, the contribution of these amino acid residues was 2.74 and 4.75 in WP hydrolysate.

Selected compounds interfering with the catalytic activity of metal ions could affect the peroxidative process, and therefore, measuring the chelating activity of compound is important for evaluating its antioxidant activity (Xia et al. 2012). Amino acids and proteins have been reported as water-soluble antioxidants because of its chelating effect on metal ions (Sakanaka and Tachibana 2006).

YP hydrolysate showed much higher Fe^2+^ chelating activity than WP hydrolysate. The level of Fe^2+^ chelating activity of YP and WP hydrolysates reached 549.87 and 289.77 μg Fe^2+^ mg^−1^, respectively.

As a result of chelating property, phosvitin shows strong antioxidant activity. Antioxidant activity of phosvitin oligopeptides derived from tryptic hydrolysate was determined by Xu et al. (2007). When the FeCl_2_ concentration was lower than 60 μmol, the chelating rate of oligopeptides at dose 100 μg/mL was 100%. Egg-yolk protein hydrolysates possessed much greater chelating ability than that of many other plant and animal origin-protein hydrolysates. For example, Chang et al. (2007) reported chelating ability ranging from 8% to 63% for hydrolysates derived from porcine hemoglobin at 5.0 mg/mL assay concentration. On the other hand, alcalase hydrolysates of sweet potato protein exhibited Fe^2+^ chelating ability, which reached 50% at 1.54 mg/mL (Zhang et al. 2012).

Our results confirmed that egg-yolk protein hydrolysates (main component of yolk protein by-product was phosvitin (data not shown)) exhibited strong chelating activity.

The above results demonstrated that the type of substrate used as well as the DH are a key factors in determining biological activity of egg protein by-products hydrolysates.

### Functional properties

The functional properties of proteins and their hydrolysates are of great importance in the fields of food and cosmetic industry. It has been observed that proteolytic degradation of food proteins affects the functional properties of the hydrolysates (Panyam and Kilara 1996; Diniz and Martin 1997; Adebiyi et al. 2008).

**Figure 8:**
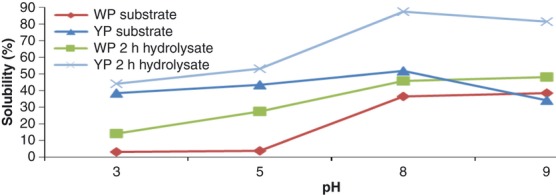
Water solubility profiles of egg-white (WP) and egg-yolk (YP) protein hydrolysates.

**Figure 9:**
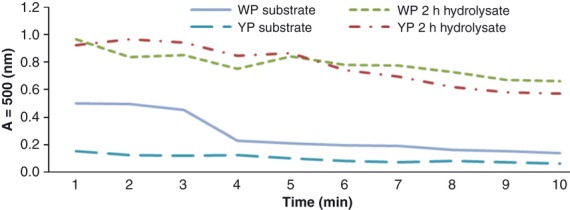
Emulsifying capacity of egg-white (WP) and egg-yolk (YP) protein preparation hydrolysates at pH 6.5.

The objective of this study was also to study the influence of the enzymatic hydrolysis of denaturated egg-yolk and egg-white protein by-products on the functional properties. The study focused on the solubility, water-holding, and emulsion capacity characterizations of the hydrolysates. Significant improvement of functionality as a result of protein hydrolysis is the increase in solubility (Panyam and Kilara 1996; Chrzanowska 1998). In this study, the solubility of the WP and YP hydrolysates obtained with Neutrase increased in comparison with the WP and YP protein preparations (Fig. 8). It has been suggested that an increase in the solubility of protein hydrolysates over that the original protein is due to the reduction of its secondary structure, and also to the enzymatic release of smaller polypeptide units from the protein (Diniz and Martin 1997). At pH 8–9, solubility of the analyzed hydrolysates was the highest. The value of soluble protein in YP and WP hydrolysates reached 87.6% and 48.3%, respectively. Similar results were obtained by Panyam and Kilara (1996), who conducted the hydrolysis of soy-protein isolate with trypsin or Alcalase, which resulted in more water-soluble peptide products than hydrolysis with chymotrypsin or rennet. Also, proteolysis of fish and seal proteins with Alcalase resulted in highly water-soluble hydrolysates (Diniz and Martin 1997). The solubility significantly affects the functional properties like gelling, emulsion, and foaming capacity. Small peptides which exhibit weak amphipathic properties destabilize emulsions. Turgeon, Gauthier, and Paquin reported that extensive hydrolysis dramatically reduces the emulsifying activity of obtained products (Turgeon et al. 1991). The hydrolysates of WP and YP showed weak emulsifying capacity (Fig. 9). No significant differences were observed in values of emulsifying capacity between obtained hydrolysates and unhydrolyzed by-products.

Water-holding capacity was increased for the Neutrase-hydrolyzed WP and YP (Table 3). The highest water-holding capacity, which reached 43.2, was observed for WP hydrolysate. Many authors observed an inverse correlation between solubility and water-holding capacity. The high solubility decreased the water-holding capacity (Diniz and Martin 1997). This is in line with our results.

## Conclusion

As new functional bioactive hydrolysates from ethanol-denaturated protein by-products left during the course of isolating lecithin from egg yolk (YP) and lysozyme and cystatin from egg white (WP), hydrolysates exhibited significant radical scavenging activity on DPPH free radicals, the ability to reduce the oxidation state of iron (III), and ability to chelate iron (II). The antioxidant activity of YP was significantly stronger than that of WP. Hydrolysates obtained by hydrolyzing YP and WP with Neutrase also effectively inhibit an ACE action in vitro. In this study, we have also shown that the enzymatic hydrolysis enhance functionalities, such as solubility and water-holding capacity. WP hydrolysate exhibited the strongest water-holding capacity.

This study indicated that YP and WP hydrolysates could be used in functional foods as natural additives with antioxidant and antihypertensive activities and with desirable functional properties.
